# Plantar forefoot pain: ultrasound findings before and after treatment with custom-made foot orthoses

**DOI:** 10.1007/s11547-021-01354-8

**Published:** 2021-04-21

**Authors:** Domenico Albano, Carlo Bonifacini, Stefania Zannoni, Susan Bernareggi, Carmelo Messina, Massimo Galia, Luca Maria Sconfienza

**Affiliations:** 1 IRCCS Istituto Ortopedico Galeazzi, Milano, Italy; 2 Sezione di Scienze Radiologiche, Dipartimento di Biomedicina, Neuroscienze e Diagnostica Avanzata, Università di Palermo, Palermo, Italy; 3 Italian Society of Medical and Interventional Radiology (SIRM), SIRM Foundation, 20122 Milan, Italy; 4 Foot and Ankle Surgery, IRCCS Istituto Ortopedico Galeazzi, Milan, Italy; 5 Scuola di Specializzazione in Radiodiagnostica, Università Degli Studi di Milano, Milan, Italy; 6 Dipartimento di Scienze Biomediche Per la Salute, Università Degli Studi di Milano, Milano, Italy

**Keywords:** Metatarsalgia, Bursitis, Morton’s neuroma, Ultrasound, Orthoses

## Abstract

**Purpose:**

No prior studies investigated the role of ultrasound in the assessment of response of patients undergoing treatment of metatarsalgia with custom-made orthoses. Our aim was to describe ultrasound findings of patients with plantar forefoot pain treated with custom-made foot orthoses.

**Methods:**

Twenty patients (15 females; mean age: 62.6 ± 11 years) affected by metatarsalgia in 27/40 feet underwent clinical evaluation before, three months and six months after treatment with custom-made full foot insole with a support proximal and an excavation below the painful metatarsals. Ultrasound was performed before and three months after the use of orthoses to examine the presence of intermetatarsal/submetatarsal bursitis, metatarsophalangeal joints effusion, anterior plantar fat pad oedema, flexor tendinitis/tenosynovitis, and Morton’s neuroma. Outcome measures were clinical response with Foot Function Index (FFI)/Visual Analogue Scale (VAS) and ultrasound features changes.

**Results:**

Median VAS and FFI before treatment were 8[5–8.5] and 45.85[32.4–59.4], respectively. After 3 and 6 months of insoles use, both median VAS (2.5 [0–5] and 0 [0–2.75], respectively) and median FFI (7.9 [3.95–20] and 0 [0–3.95], respectively) showed a significant reduction in pain and disability (p < .001). Before treatment, ultrasound revealed 22 intermetatarsal bursitis, 16 submetatarsal bursitis, 10 joint effusions, 20 fat pad oedema, 3 flexor tendinitis/tenosynovitis and 3 Morton’s neuromas. After 3 months of treatment, a significant decrease of intermetatarsal bursitis (7, *p* < .001) was observed. No significant changes were observed in any other ultrasound parameters.

**Conclusion:**

Ultrasound might be able to detect some imaging features associated with the response of forefoot pain to custom-made foot orthoses, especially intermetatarsal bursitis.

## Introduction

Metatarsalgia is one of the most frequent painful conditions of the forefoot [1]. Patients usually present with forefoot plantar pain during weight-bearing activities, typically in the area across the second through the fourth metatarsal heads. Metatarsalgia typically develops secondary to mechanical disorders that lead to high-impact loading under the metatarsal heads, but it can also originate from trauma, instability of the metatarsophalangeal joints, inflammatory or infectious processes, and bone tumours [2, 3]. Metatarsalgia may be treated either conservatively (use of metatarsal pads or bars, physical therapy, steroid or alcohol injection, radiofrequency ablation) [4, 5] or surgically [6, 7], according to its main cause*.* The first step is generally appropriate footwear with custom-made or prefabricated insoles to reduce plantar pressure and subsequent pain in the metatarsal head region [7, 8]. Insoles have been shown to reduce post-fatigue loading under the toes and the midfoot, with custom-made insoles being able to further decrease loading under the heel during running when compared with prefabricated insoles [8]. Weight-bearing radiographs, ultrasound, and magnetic resonance imaging can all be used to reach the diagnosis to guide treatment [9–18]. Foot and ankle ultrasound has been increasingly used as a diagnostic tool with the ability to dynamically evaluate during motion and direct palpation [19, 22]. Most of the causes of metatarsalgia can be diagnosed by forefoot ultrasound, including bursitis, Morton’s neuroma (MN), tendinitis/tenosynovitis, and neoplastic masses. According to the last clinical guidelines for musculoskeletal ultrasound by the European Society of Musculoskeletal Radiology, ultrasound is recommended as the first-choice imaging modality for both ankle/foot tendinopathies and MN [23, 24]. However, no previous studies have investigated how ultrasound features change over time after conservative treatment of metatarsalgia. Thus, the aim of this study was to describe ultrasound findings of patients with plantar forefoot pain treated with custom-made foot orthoses.

## Materials and methods

### Study design

This retrospective study evaluated ultrasound findings of patients with metatarsalgia managed at the Podiatry Clinic, the Unit of Diagnostic and Interventional Radiology and the Foot and Ankle Surgery department of our Institution from December 2017 to October 2018. Our study included a consecutive cohort of patients with metatarsalgia examined clinically and using ultrasound at baseline and three months after conservative treatment with a custom-made full foot insole with proximal support and excavation below the painful metatarsals. All patients were then clinically re-evaluated after 6 months as a routine clinical procedure at our Institution. We included patients ≥ 18 years of age, with metatarsalgia of central metatarsal heads who presented for initial evaluation for localized pain in the forefoot (second, third, and fourth metatarsals and their respective metatarsophalangeal joints) made worse with weight bearing. Exclusion criteria were known autoimmune and rheumatic diseases, behavioural and mental disorders, pregnancy, recent trauma, neurodegenerative diseases, and systemic neuropathies. This study was approved by our Institutional Review Board, with a waiver for patients’ informed consent.

### Clinical assessment and orthoses construction

**Fig. 1:**
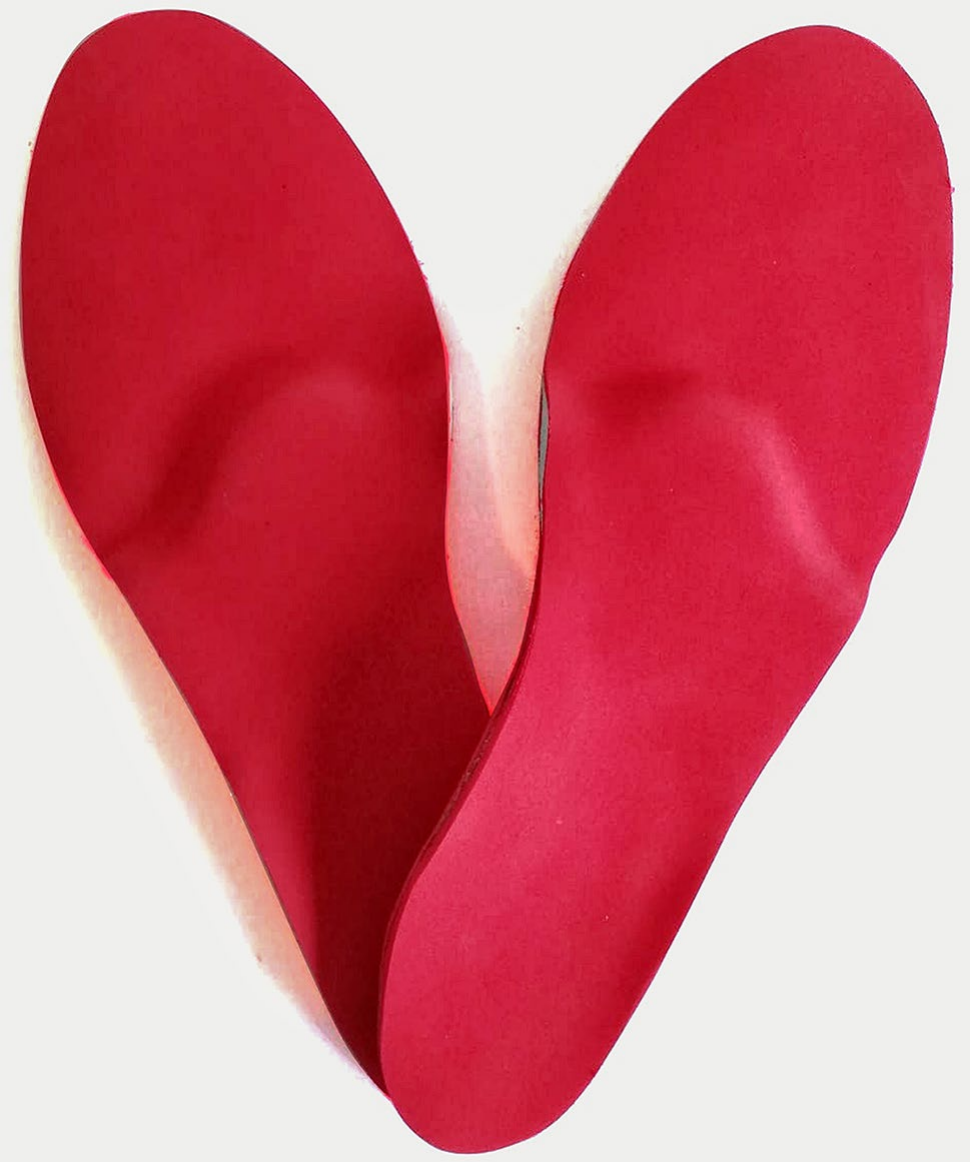
The orthotics were made placing on an expanded ethylene vinyl acetate material a retrocapped latex bar at the level of the metatarsal heads and a polypropylene support of the longitudinal medial vault

Every patient first underwent clinical assessment by both an experienced orthopedist and a podiatrist. The diagnosis of metatarsalgia was made when pain in the forefoot area with increased stress over the metatarsal head region was observed. A general clinical examination was performed to assess any possible cause of metatarsalgia. Standard clinical examination included the following steps. Initially, the foot was inspected for skin integrity, swelling, and temperature. Then, a biomechanical examination of the tibiotarsal, subastragalic, mediotarsal, Lisfranc, and metatarsophalangeal joints was performed, to evaluate the range of motion of every joint, in order to exclude the presence of joint stiffness. The presence of deformities of the toes, their mobility, and possible subluxations were analyzed. Specific attention was paid to the first ray to determine its position (neutral, dorsiflexed, or plantarflexed) and correct mobility. The medial longitudinal arch, the alignment of the hindfoot, the forefoot, and their relationship were examined. The plantar fascia was palpated to assess plantar fasciitis and the course of the Achilles tendon to assess any painful areas. To investigate the presence of bursitis, acupressure of metatarsal heads and intermetatarsal spaces was applied and the Mulder test was performed to assess the presence of a MN. Finally, the patient was asked to walk barefoot to examine the movement of the foot during the gait cycle, the correct support of the heel and forefoot, and to assess the presence of asymmetries during the gait. The Visual Analog Scale (VAS) was used to measure subjective pain experienced by the patient [25]. Foot symptoms were determined using the Foot Function Index (FFI) [26], a validated patient-administered index composed of 23 questions divided into three groups, developed to measure the impact of foot disease on its function in terms of pain, disability, and activity restriction. All patients were treated with orthotics made on a two-dimensional impression, placing on an expanded ethylene vinyl acetate material a retrocapped latex bar located 6.5 mm proximally to the second metatarsal head and a polypropylene support of the longitudinal medial vault (Fig. 1). Every patient was advised to wear sneakers with a heel support. No other treatments were used on these patients.

### Ultrasound evaluation

Baseline and 3-months ultrasound examination was performed by the same operator, a senior radiologist with 10 years experience in musculoskeletal ultrasound. All examinations were performed with the same linear transducer (5–14 MHz, Preirus 400, Hitachi, Japan). During the ultrasound examinations, patients were laying supine on the examination bed with the ankle relaxed. A dorsal approach was performed to identify pathological effusion in the metatarso-phalangeal joints. As a small amount of metatarsophalangeal joint effusion is physiologic, we considered this finding pathologic when an increase in intra-articular fluid was observed in at least one metatarsophalangeal joint when compared with the other ipsilateral and contralateral ones. A plantar approach was used to detect the presence of: intermetatarsal bursitis as a hypo to anechoic fluid collection in the intermetatarsal space, indeed, intermetatarsal bursa is not visible with ultrasound in normal conditions [27]; adventitious submetatarsal bursitis as a compressible hypo to anechoic fluid collection beneath the metatarsal heads [28]; MN as a round or peanut-shaped hypoechoic nodule placed in the plantar aspect of the intermetatarsal space being more visible squeezing the metatarsals during the scan [29]; flexor tendinitis as thickening/thinning of flexor tendons and flexor tenosynovitis as increased fluid content within tendons sheath [30]; oedema of the anterior plantar fat pad that has been scarcely reported in literature, although in our experience is frequently encountered in patients with metatarsalgia. As mentioned, the evaluation of the intermetatarsal spaces was dynamically performed in coronal and sagittal planes performing the Mulder manoeuvre to increase diagnostic sensitivity for MN. This evaluation was done scanning the plantar surface of the forefoot, applying pressure on the dorsal aspect of the intermetatarsal space with a finger not involved in probe holding and clasping the metatarsal heads with the fingers of the left hand [31]. The plantar fascia was also checked to exclude pathologic conditions of the fascia potentially determining metatarsalgia including plantar fasciits (as a hypoechoic appearance and thickening of more than 4 mm of the fascia), plantar tear (as a partial or complete disruption of plantar fibres), and plantar fibromatosis (as single or multiple eccentric and hypoechoic nodular thickenings of the fascia) [32]. Further, the ultrasound examination included the evaluation of metatarsal bony profiles to search for signs of stress fractures including cortical lines and callus formation [29].

### Statistical analysis

VAS and FFI, together with ultrasound findings, were used for baseline evaluation and to monitor the response to treatment in follow-up examinations. Continuous variables are reported as mean ± standard deviation. Discrete variables are summarized as median and interquartile range. Proportions are expressed as percentages. Frequencies were compared using the McNemar test. Paired t test for parametric continuous data was also used. Statistical analysis was performed using SPSS^®^ software (v. 25, IBM, Armonk, New York, NY). A P value lower than 0.05 was considered statistically significant [33].

## Results

### Clinical findings

**Fig. 2:**
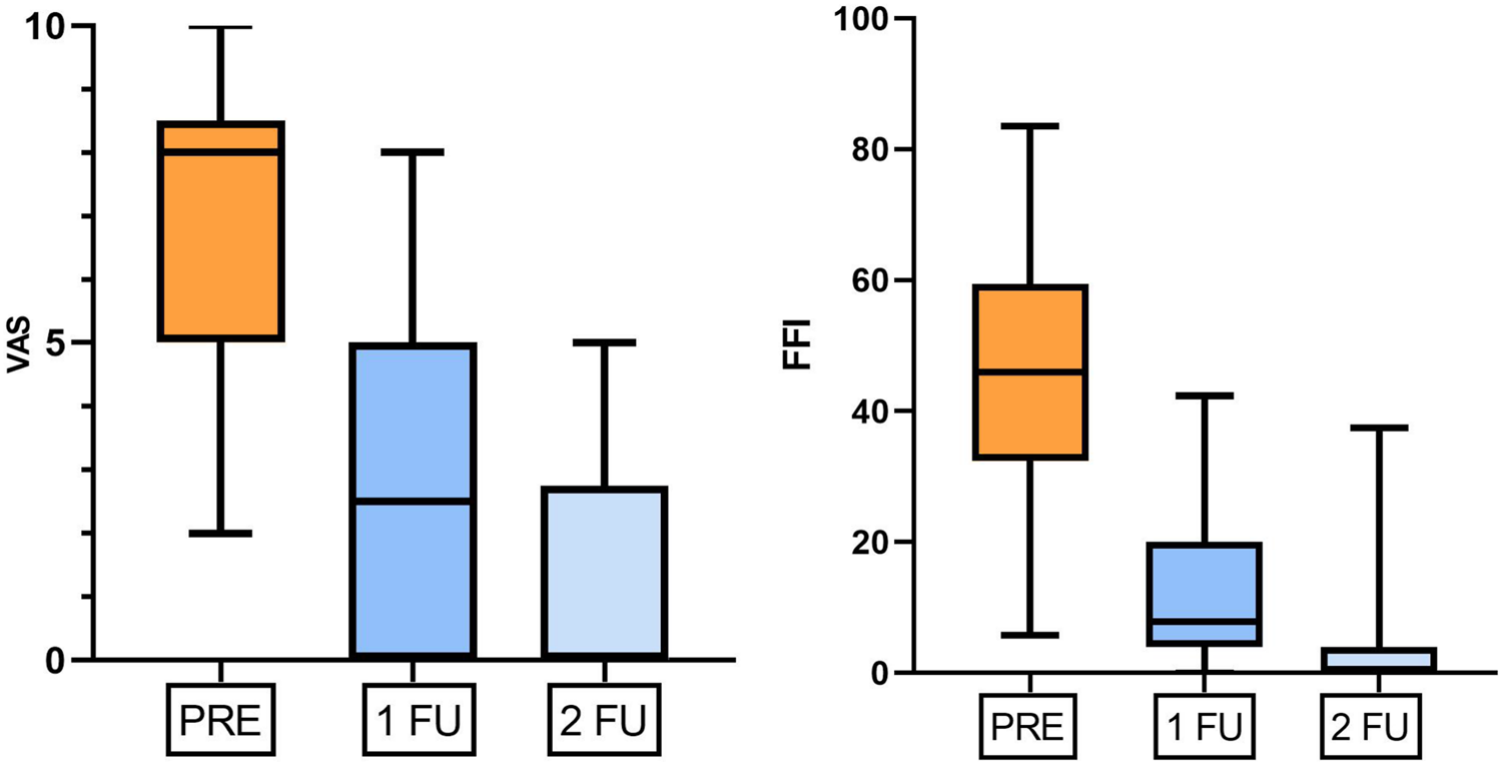
Boxplots show the progressive and significant reduction of both median VAS score and the median FFI score from baseline to 3 months (1FU)  and 6 months (2FU) evaluation (*p* < .001)

A total of 20 patients (15 women, 5 men; mean age: 62.6 ± 11 years; range 36–78 years) with forefoot pain in 27 out of 40 feet were included in this study, with seven of them having bilateral pain. The median VAS score and the median FFI score before treatment were 8 [interquartile range = 5–8.5] and 45.85 [32.4–59.4], respectively. After 3 and 6 months of insoles use, both median VAS (2.5 [0–5] and 0 [0–2.75], respectively) and median FFI (7.9 [3.95–20] and 0 [0–3.95], respectively) showed a significant reduction in pain and disability (*p* < 0.001) (Fig. 2).

### Ultrasound findings

**Fig. 3:**
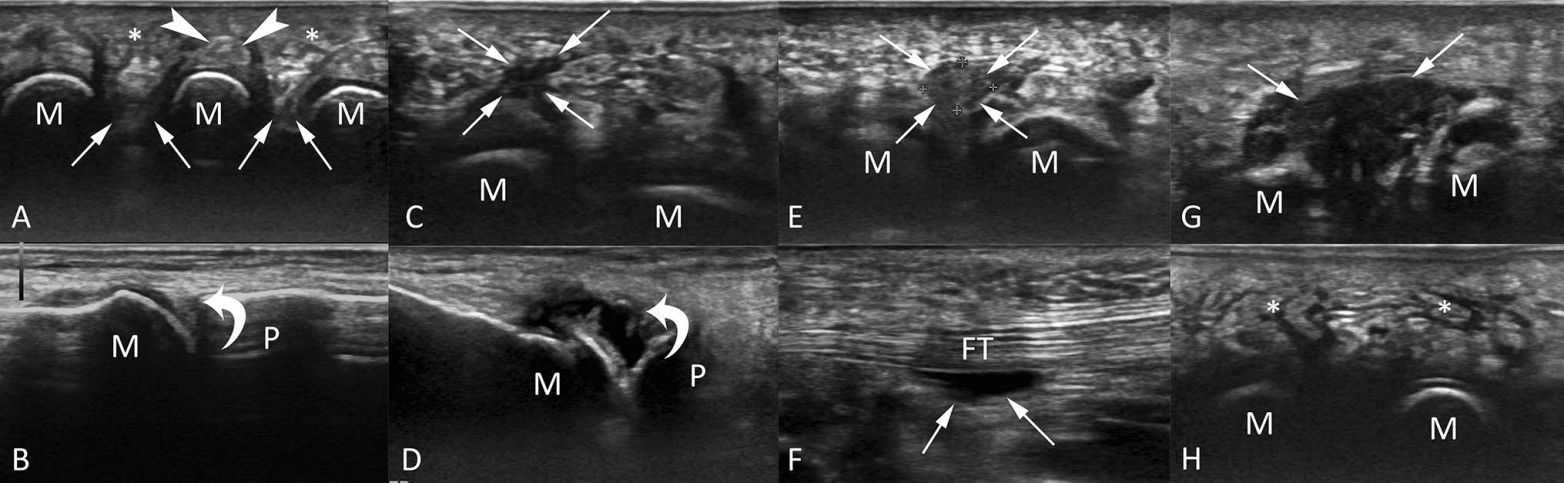
Ultrasound findings of some representative cases from our study population. Transverse image with plantar approach **a** of a patient with normal anterior plantar fat pad (asterisks), flexor tendon (headarrows), and intermetatarsal spaces (arrows). Longitudinal image with dorsal approach **b** of the first metatarsophalangeal joint with no effusion (curved arrow). Transverse image with plantar approach of submetatarsal bursitis (**c**, arrows). Longitudinal image with dorsal approach **d** of the first metatarsophalangeal joint with pathological effusion (curved arrow). Transverse image with plantar approach of Morton’s neuroma (**e**, arrows). Longitudinal image with plantar approach of flexor tendinitis (**f**, arrows). Transverse image with plantar approach of intermetatarsal bursitis (**g**, arrows). Transverse image with plantar approach **h** of a patient with oedema of the anterior plantar fat pad (asterisks). M = metatarsal; P = proximal phalange; FT = flexor tendon

Intermetatarsal bursitis was observed in 81% of feet (22/27) at baseline ultrasound and in 25% (7/27) at three months, with a statistically significant reduction of this imaging feature (*p* < 0.001). At baseline ultrasound, 59% of feet (16/27) showed submetatarsal bursitis, 37% (10/27) joint effusion, 74% (20/27) oedema of the anterior plantar fat pad. After three months of conservative therapy, we noted a reduced frequency of these ultrasound findings, but without statistically significant differences from baseline ultrasound (*p* > 0.06). Further, both MN and flexor tendinitis/tenosynovitis were observed in 11% of feet (3/27) at baseline ultrasound, with no improvement at 3-months sonographic examination. Neither pathologic findings of the plantar fascia nor signs of stress fractures were observed in our series. All ultrasound findings before and after three months of conservative treatment are shown in Table 1. Figure 3 shows the ultrasound findings of some representative cases from our study population.

**Table 1:** All ultrasound findings before and after three months of conservative treatment

	Pre-treatment	Post-treatment	*p* value
Intermetatarsal bursitis	81% (22/27)	25% (7/27)	< 0.001*
Submetatarsal bursitis	59% (16/27)	37% (10/27)	0.074
Joint effusion	37% (10/27)	18% (5/27)	0.065
Fat pad imbibition	74% (20/27)	51% (14/27)	0.074
Morton’s neuroma	11% (3/27)	11% (3/27)	> 0.999
Tendinitis/tenosynovitis	11% (3/27)	11% (3/27)	> 0.999

*Indicates a *P* value lower than 0.05, which was considered as statistically significant

## Discussion

Our main finding was that some ultrasound features seem to be associated with the response of metatarsalgia to custom-made foot orthoses, particularly the decrease of intermetatarsal bursitis. Conversely, we did not observe MN and flexor tendinitis/tenosynovitis changes after conservative treatment of forefoot pain.

The use of orthotics in patients affected by forefoot pain has a proven beneficial effect on pain relief. This is probably related to the reduction in pressure on the metatarsal heads, which invariably increases with age. Indeed, in older people, plantar fat pads show greater stiffness, dissipate more energy when compressed and are slower to recover after the load is removed. This explains the advanced mean age of patients included in our series (62.6 years) [34]. In daily practice, metatarsal head unloading is the main strategy to prevent pressure sores and to reduce forefoot pain [35]. In 2018, a systematic review stated that the use of custom-made foot orthoses improves the level of forefoot pain in patients affected by rheumatoid arthritis, hallux abductus valgus and secondary metatarsalgia due to the increase in soles pressures [36–38]. To date, there is no strong evidence regarding the superiority of using custom-made insoles compared with prefabricated ones in patients affected by metatarsalgia [8]. In our study, the use of custom-made foot orthoses was associated with decrease in pain and disability in patients with metatarsalgia, as shown by the significant reduction of the VAS and FFI scores after treatment, but this result should be supported by randomized controlled trials to be confirmed.

In the literature, several different tools and instruments are used to evaluate the results of a conservative treatment in patients affected by metatarsalgia. Postema et al. used plantar pressure measurements as outcome markers after orthotics use [35]. Mejjad et al. used gait analysis to evaluate the results after one month of foot orthotics use [39]. Ultrasound scan is now considered, together with standard radiograph, as a first-step examination for forefoot disorders [23, 40, 41], although its use to monitor patient’s outcome after therapy has not been validated yet. To our knowledge, this is the first study focused on the description of ultrasound features before and after conservative treatment of forefoot pain. After three months of foot orthotics treatment, we observed a statistically significant reduction in the number of intermetatarsal bursitis detected by ultrasound. Intermetatarsal bursitis often determines metatarsalgia, especially during walking, tingling and numbness, also mimicking MN. Small fluid collections with maximum transverse diameter of 3 mm in the intermetatarsal space are considered physiologic in MRI [42], although intermetatarsal bursa is not visible with ultrasound in normal conditions [27]. In pathologic conditions, intermetatarsal bursae become cystic-like lesions that can show synovium thickening and present as compressible hypoechoic structure on dynamic ultrasound, which differentiates it from a MN that is not compressible [29]. In our study, intermetatarsal bursitis was the most frequent ultrasound feature, besides being the only one presenting significant reduction after 3 months of custom-made foot orthoses. It should be noted that the frequency of intermetatarsal bursitis (81%) was quite higher than that obtained by Iagnocco et al. [30] in 2001, where the authors found this ultrasound feature in 23% of patients with metatarsalgia. This relevant difference might have several explanations. First, we can postulate that advancements in ultrasound technology [43] could have helped us to identify small fluid effusion in the intermetatarsal spaces, as well as to differentiate them from neuromas. Then, the small sample size may have affected these data, since Iagnocco et al. evaluated 112 patients with metatarsalgia. Further, the authors did not report the experience of the operators who performed ultrasound examination that should be taken into account in this setting, since small intermetatarsal collections may be missed by less experienced operators. Our results might turn the spotlight on the role of the expansion of the intermetatarsal bursae as possible primary pain generators in patients with forefoot pain. The association of intermetatarsal bursitis and forefoot pain has been scarcely reported in the literature and no previous papers have discussed the association of disappearance/reduction of intermetatarsal bursitis and clinical response to conservative treatment of metatarsalgia. To date, the crucial role of intermetatarsal bursitis has been postulated only in rheumatological disorders of the foot rather than in biomechanical forefoot disorders [44]. Thus, these results need to be confirmed by randomized controlled studies and could open non-negligible diagnostic and therapeutic possibilities (e.g. infiltrative treatment of intermetatarsal bursitis).

On the other hand, a statistically significant reduction of adventitious submetatarsal bursitis, joint effusion, and oedema of anterior plantar fat pad was not observed. This could be also related to the relatively small sample size. Submetatarsal adventitious bursae, as opposed to intermetatarsal ones, have no walls with mesothelial tissue [45]. Submetatarsal bursitis is often observed in the anterior plantar fat pad at the sites of higher pressure and friction, especially at the level of the first and fifth metatarsal head, presenting at ultrasound as a compressible hypoechoic structure ovoid in shape within an oedematous fat pad [29.46]. On the other hand, no improvement of the number of flexor tendinitis/tenosynovitis and MN was observed. Probably, flexor tendinopathy plays a minor role in forefoot pain if compared with other potential pain generators. Moreover, we should underline that not all MN are symptomatic, indeed those larger in size [42] and with plantar extension [47] tend to be more symptomatic. This could explain why those patients with MN had pain relief despite the obvious persistence of MN itself after conservative treatment. Notably, it should be considered that MN can still be space-occupying, while its symptoms subside by decreasing oedema around the neuroma. A possible explanation for the discrepancy between clinical results and ultrasound findings after treatment may be the early timepoint of the second ultrasound examination, carried out only three months after the use of the orthoses, a time that could have been insufficient to determine the sonographic reduction of inflammation.

The present study has some limitations that should be pointed out. First, as already mentioned, the relatively small sample size, therefore, the generalization of our results to routinely encountered patients during clinical practice needs to be confirmed in a larger series of patients. Second, longer follow-up could help to understand how ultrasound features change over time after conservative treatment. The short time of follow-up can explain the difference between clinical benefit after orthotic therapy and the ultrasound appearance at 3-months examination. Further studies with longer follow-up are needed to confirm our hypothesis. Then, we did not include healthy subjects as controls and we did not randomize our patients in treatment arms, thus prospective randomized controlled studies should be done to understand how ultrasound features change from responding to non-responding patients. Last, we had no surgical and histopathological proof of the intermetatarsal masses (MN and intermetatarsal bursitis) described by ultrasound, which was performed by only one experienced radiologist, thus no comparison was done with a second operator.

In conclusion, we have reported the ultrasound features that can be observed in patients with metatarsalgia before and after conservative treatment. Ultrasound might be able to detect some imaging features associated with the response of forefoot pain to custom-made foot orthoses, especially identifying sonographic changes of intermetatarsal bursitis, while no changes of MN and flexor tendinitis/tenosynovitis seem to occur in responding patients after conservative treatment. This study might help to correctly describe the ultrasound picture of patients suffering from metatarsalgia.
